# Clinical significance of atypical protein kinase C (PKCι and PKCζ) and its relationship with yes-associated protein in lung adenocarcinoma

**DOI:** 10.1186/s12885-019-5992-7

**Published:** 2019-08-14

**Authors:** Kyung-Hee Kim, Chaeuk Chung, Jin-Man Kim, Dahye Lee, Sang Yeon Cho, Tae Hee Lee, Hyun Jin Cho, Min-Kyung Yeo

**Affiliations:** 1 Department of Pathology, Chungnam National University School of Medicine, Munwha-ro 266, Jung-gu, Daejeon, 35015 Republic of Korea; 2 Division of Pulmonology, Department of Internal Medicine, College of Medicine, Chungnam National University, Daejeon, 35015 Republic of Korea; 3 School of Medicine, Chungnam National University, Munwha-ro 266, Jung-gu, Daejeon, Republic of Korea; 4 The Biobank of Chungnam National University Hospital, Munwha-ro 282, Jung-gu, Daejeon, Republic of Korea; 5 Department of Thoracic Surgery, College of Medicine, Chungnam National University, Daejeon, 35015 Republic of Korea

**Keywords:** Atypical protein kinase C, Protein kinase C iota, Protein kinase C zeta, Lung adenocarcinoma

## Abstract

**Background:**

Protein kinase C iota (PKCι) and protein kinase C zeta (PKCζ) are two atypical protein kinase (aPKC) enzymes that contribute to cell proliferation and cancer development. The Hippo/YAP pathway is commonly disrupted and upregulated in cancers. Herein, the expression patterns and clinical relevance of PKCι and PKCζ are evaluated in relation to YAP, a downstream effector of Hippo, in lung adenocarcinoma (LAC). The protein and mRNA expression levels of PKCι, PKCζ, YAP, and their phosphorylated forms, namely p-PKCι, p-PKCζ and p-YAP, are evaluated in relation to clinicopathological factors, including patient survival.

**Methods:**

A total of 200 primary LAC tissue samples were examined by immunohistochemistry for PKCι, p-PKCι, PKCζ, p-PKCζ, YAP, and p-YAP protein expression. Sixty pairs of LAC and non-neoplastic lung tissue samples were assessed for PRKCI, PRKCZ, and YAP mRNA levels. PKCι, p-PKCι, PKCζ, and p-PKCζ protein expression were evaluated by Western blot analysis in the PC9 and PC9/GR LAC cell lines with YAP modulation.

**Results:**

LAC demonstrated cytoplasmic PKCι, p-PKCι, PKCζ, and p-PKCζ immunostaining patterns. Positive aPKC protein expressions were related with poor patient survival. Especially, increased p-PKCι protein expression was significantly correlated with higher pathological stage and shortened overall survival. YAP overexpression contributes phosphorylation of PKCι and PKCζ protein expression in the LAC cell line.

**Conclusions:**

PKCι and PKCζ are related to YAP in LAC. PKCι and PKCζ play distinct roles in LAC; specifically, p-PKCι overexpression is suggested to underlie factors that indicate a poor prognosis.

**Electronic supplementary material:**

The online version of this article (10.1186/s12885-019-5992-7) contains supplementary material, which is available to authorized users.

## Background

Protein kinase C (PKC) refers an isoenzyme family of serine/threonine kinases that was originally identified as a cellular receptor in 1983 [1]. PKC isozymes are ubiquitously expressed in various human cell types, and PKCs are thought to carry out distinct and non-redundant functions [2]. The PKC family is categorized as classical (α, β, γ), novel (δ, ε, η, θ), and atypical (ζ, λ, ι, μ) based on its second messenger requirements. The atypical PKC (aPKC) isoforms are independent of calcium and diacylglycerol, in contrast to other PKCs [3]. aPKCs have been shown to function in cell polarity, differentiation, migration, and proliferation [4, 5]. aPKCs also play a pivotal role in tumor progression, tumor metastasis, and patient survival in the context of many cancers [6, 7]. The inhibition of protein kinases has been examined for cancer treatment, and aPKC inhibition is worthy of evaluation as a potential candidate therapeutic strategy [8].

Lung cancer, the most common cause of cancer death in the United States, requires novel target agents for advanced cancer patients [9]. Lung adenocarcinoma (LAC) is the most common type of lung cancer and is most likely to occur in young people. Understanding the aPKC underlying LAC can help to identify a therapeutic target for LAC. Up- and downregulation of aPKC have been demonstrated in several cancers; however, the exact mechanism of aPKC involvement is still unclear. Activation of aPKC depends on protein-protein interactions and requires downstream effectors [3]. aPKC promotes cell junction formation and plays a critical role in cell polarity [10]. Upstream regulators of the Hippo pathway are thought to be linked to cell polarity complexes [11]. The Hippo/YAP pathway is occasionally disrupted, and nuclear accumulation of YAP has been reported to be associated with tumor aggressiveness in LAC [12]. aPKC activity results in deregulation of Hippo/YAP signalling and induces transformed epithelial cell growth, suggesting the possibility of a relationship between aPKC and the Hippo/YAP pathway in LAC [13].

To investigate the role of aPKC and Hippo/YAP signalling in LAC, atypical protein kinase iota (PKCι) and atypical protein kinase zeta (PKCζ), which belong to the aPKC isoenzyme subgroup, were evaluated in relation to YAP, a downstream effector of Hippo. Herein, PKCι, PKCζ, and YAP expression and the levels of the phosphorylated forms of p-PKCι, p-PKCζ, and p-YAP were assessed in LAC tissue samples. The expression patterns of PKCι, PKCζ, and YAP were assessed at the protein and mRNA levels and were analysed in relation to clinicopathological features, including LAC patient survival.

## Methods

### Patients and tissue samples

A total of 200 paraffin-embedded LAC tissue samples were obtained from 200 patients who underwent surgical treatment and were histologically diagnosed with LAC at the Chungnam National University Hospital (Daejeon, South Korea) from January 2008 to December 2017. In a surgical specimen, the most representative and viable tumor area was selected and marked on the haematoxylin and eosin (H&E)–stained slides. To construct a tissue microarray, tissue columns (3.0 mm in diameter) were punched from the original paraffin blocks and inserted into new recipient paraffin blocks (each tissue columns containing 30 holes). Patients overall survival (the length of time from the date of diagnosis to the date of death), disease-free survival (the length of time from the date of diagnosis to the date of identification of recurrence), pre- or post-surgical chemotherapy, and radiotherapy history were reviewed by two pathologists (K.H.K and M.K.Y.) for identification of clinicopathological features. Patients who received pre-operative chemotherapy were excluded from this study. LAC stages were determined according to the American Joint Committee on Cancer Staging System, eighth edition [14]. This study protocol was approved by the Institutional Review Board of Chungnam National University Hospital and complied with the tenets of the Declaration of Helsinki (CNUH 2016–08-060). The study was retrospective, and a waiver of consent was approved by the Institutional Review Board.

### Immunohistochemical staining analysis

Two hundred samples were cut from the tissue microarray paraffin blocks. Tissue sections on the coated microslides were deparaffinized with xylene and hydrated in serial solutions of alcohol. The sections were heated in a pressure cooker (containing 10 mmol/L sodium citrate (pH 6.0)) for 3 min for antigen retrieval. Endogenous peroxidase blocking was performed using 0.03% hydrogen peroxide for 10 min. The sections were incubated overnight at 4 °C with the following primary antibodies: rabbit polyclonal anti-YAP antibody (1:200, #4912, Cell Signaling Technology, Danvers, MA, USA), rabbit polyclonal anti-phosphorylated-YAP (p-YAP) antibody (phospho Ser127) (1:100; Cell Signaling Technology, Danvers, MA, USA), mouse monoclonal anti-PKCι antibody (1:50, #610175, BD Transduction Laboratory, Lexington, KY, USA), rabbit polyclonal anti-phosphorylated-PKCι (p-PKCι) antibody (phospho T555 + T563) (1:100, ab5813, Abcam, Cambridge, UK), rabbit polyclonal anti-PKCζ antibody (C-20) (1:300, sc-216, Santa Cruz Biotechnology, Santa Cruz, CA, USA), and mouse monoclonal anti-phosphorylated-PKCζ (p-PKCζ) antibody (H-2) (phospho T410) (1:200, sc-271,962, Santa Cruz Biotechnology, Santa Cruz, CA, USA). After washing, samples were incubated in Dako REAL EnVision/horseradish peroxidase rabbit/mouse detection reagent for an additional 20 min at room temperature followed by additional washing. After rinsing, chromogen was developed for 2 min. Slides were counterstained with Meyer’s haematoxylin, dehydrated, and topped with coverslips.

The primary antibody was omitted in the negative control samples. Mouse brain tissue was used as positive control followed by the datasheets. Four representative whole sections of the lung adenocarcinoma tissue were used to validate each antibody. Proper concentration, temperature, and time for immunohistochemistry were assessed. Four antibodies were stained diffusely and not patchy that 200 cores of samples from the tissue microarray paraffin blocks were considered to represent the whole tissue samples.

To evaluate co-expression pattern, dual immunohistochemical staining was performed using an automated immunostainer Ventana Discovery XT (Ventana Medical Systems Inc. Tucson, Arizona). A primary mouse monoclonal antibody to anti-PKCι antibody (1:100, #610175, BD Transduction Laboratory, Lexington, KY, USA) and a rabbit polyclonal anti-PKCζ antibody (C-20) (1:300, sc-216, Santa Cruz Biotechnology, Santa Cruz, CA, USA) were used. These antibodies were incubated with the sections at 31 °C for 36 min and 20 min each. After washing, samples were incubated in DAB kit (DISCOVERY UltraMap anti-Ms HRP, LOT: Y18365) and RED kit (DISCOVERY UltraMap anti-RB Alk Phos,LOT: Y28104) for an additional 32 and 20 min at room temperature followed by additional washing.

Immunohistochemical staining was scored using digitally scanned files with the ScanScope program (Aperio ScanScope CS system, Vista, CA, USA). Both the intensity of immunohistochemical staining and the proportion of stained epithelial cells in each stained slide were evaluated by Allred et al. method [15]. The proportion scores (0, 0; 1, > 0 to 1/100; 2, > 1/100 to 1/10; 3, > 1/10 to 1/3; 4, > 1/3 to 2/3; 5, > 2/3 to 1) and intensity scores (0, negative; 1, weak; 2, moderate; 3, marked) were added to obtain the total score (range: 0–8). Immunohistochemical expression was categorized as “high (expression at the median value or more)” and “low (expression at less than the medium value)”. Each sample was examined separately and scored by two pathologists (K.H.K. and M.K.Y.) who were blinded to the patients’ details. Discrepancies in scores were discussed to obtain a consensus.

### Quantitative real-time reverse-transcription polymerase chain reaction (qRT-PCR)

Sixty pairs of LAC and non-neoplastic lung tissue (more than 2 cm apart from the tumor) samples stored at − 80 °C in liquid nitrogen were obtained from the National Biobank of Korea (Chungnam National University Hospital, a member of the Korea Biobank Network) from January 2010 to December 2017. Under the review of H&E-stained frozen sections, one vial (100 mg) of LAC and non-neoplastic tissue samples were obtained. Total RNA was extracted from pairs of LAC and non-neoplastic lung tissue using a QIAGEN kit (Valencia, CA, USA) following the manufacturer’s instructions. Reverse transcription was performed with RevertAid H Minus Reverse Transcriptase (Thermo Scientific, Waltham, USA) according to the manufacturer’s instructions. Real-time PCR was performed in a Rotor-Genes Q cycler machine (Qiagen) using a Rotor-Genes SYBR Green PCR kit (Qiagen), according to the manufacturer’s instructions, in a total volume of 20 μl. The primers used for PCR amplification were as follows: (a) YAP1 (sense: 5′- tgaaaagcctcagcttgggaag − 3′, antisense: 5′- ccaacttttgccctcctcca − 3′) (b) PRKCI (sense: 5′- tgctgtttcccatagggcatt − 3′ antisense: 5′- tcgaaggccccaaaagaagtc − 3′), and (c) PRKCZ (sense: 5′- accccagacgatgaggatgc − 3′, antisense: 5′- accgactcctcggtggacag − 3′). To correlate the threshold (Ct) values from the amplification plots to copy number, a standard curve was generated, and a non-template control was run with every assay. All samples were run in duplicate, and the average value was used. The relative quantification values of YAP, PRKCI, and PRKCZ in each tissue sample were graded as low (less than the paired non-neoplastic tissue value) or high (greater than the paired non-neoplastic tissue value) for categorical analyses. Samples with insufficient RNA levels or failed PCR results were excluded.

### Cell lines and transient transfection

The human lung cancer cell lines PC9 cells (human LAC cell line harbouring the epidermal growth factor receptor (EGFR)-exon 19 deletion) and PC9/GR cells (human LAC cell line harbouring the EGFR T790 M mutation, which is resistant to EGFR-tyrosine kinase inhibitor (TKI) therapy) were cultured at 37 °C in 5% CO2 in RPMI-1640 medium (WelGENE, Daegu, Republic of Korea) containing 10% foetal bovine serum (FBS) (WelGENE, Daegu, Republic of Korea). The pDKflag-YAPWT, pDKflag-YAP2SA, and control vector plasmids were provided by Prof. Lim (KAIST, Daejeon, Republic of Korea). The transfections of the different DNA constructs were performed using Lipofectamine 2000 (Invitrogen, Thermo Scientific, Waltham, USA) according to the manufacturer’s instructions. Further assays were conducted after a 48-h incubation of transiently transfected cells.

### Western blot analysis

Cells were harvested and suspended in protein lysis buffer (Translab, Daejeon, Republic of Korea) and heated at 100 °C for 10 min. Protein concentrations were determined by means of the Bio-Rad protein assay (#500–0006, Bio-Rad, California, USA). A total of 30 μg of protein (μg/ml) was separated on a 10% SDS-PAGE gel and transferred to a polyvinylidene difluoride membrane (Millipore). The membrane was blocked with 2% dry skim milk and incubated with anti-β-actin (sc-47,778, Santa Cruz Biotechnology), anti-YAP (#4912S, Cell Signaling Technology), anti-PKCι (#610175, BD Biosciences), anti-p-PKCι (ab5813, Abcam), anti-PKCζ (sc-17,781, Santa Cruz Biotechnology), and anti-p-PKCζ (sc-271,962, Santa Cruz Biotechnology). Blots were developed using an enhanced chemiluminescence detection kit (Thermo).

### Statistical analysis

Associations between the PKCι, p-PKCι, PKCζ, p-PKCζ, YAP, and p-YAP immunohistochemical protein and relative PRKCI, PRKCZ, and YAP mRNA expression levels and selected clinicopathological variables for LAC were examined with Spearman rank correlation coefficients, Mann-Whitney *U* tests, and Kruskal-Wallis tests. The Wilcoxon signed-rank test was used for group comparisons. For the univariate analysis, overall and disease-free survival curves with log-rank and Breslow tests were generated using the Kaplan-Meier method. Multivariate survival analysis was performed using the Cox proportional hazard regression model. Statistical significance was set at *P* < 0.05 (SPSS 24.0; SPSS Inc., Chicago, IL, USA).

## Results

### Immunohistochemical expression of YAP, p-YAP, PKCι, p-PKCι, PKCζ and p-PKCζ in LAC tissue samples

**Fig. 1:**
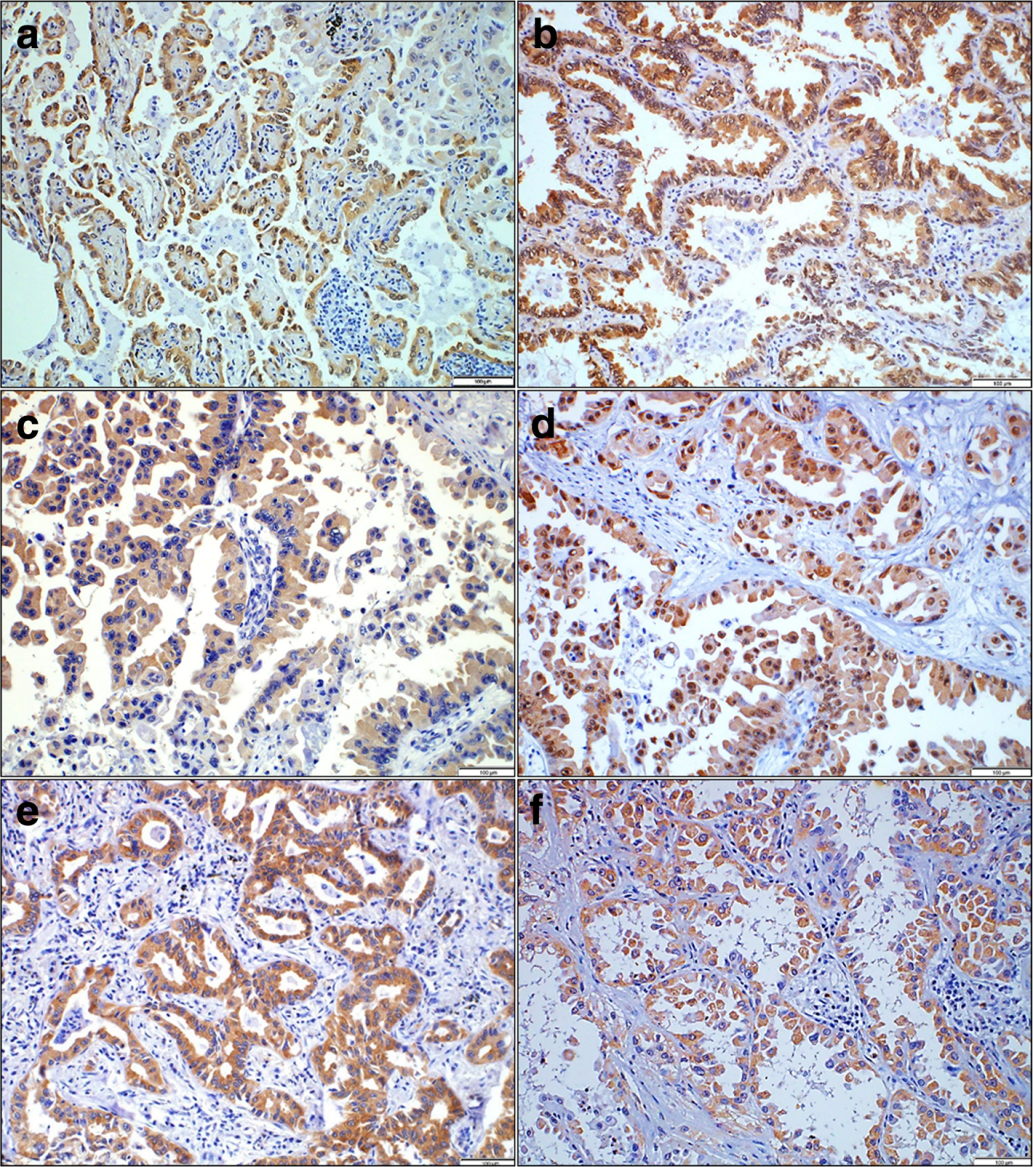
Representative immunohistochemical expression levels of (**a**) YAP, (**b**) p-YAP, (**c**) PKCι, (**d**) p-PKCι (**e**) PKCζ, and (**f**) p-PKCζ in LAC

YAP, p-YAP, PKCι, p-PKCι, PKCζ and p-PKCζ protein expression levels were evaluated using immunohistochemistry in a total of 200 LAC TMA tissue samples. Immunostaining for YAP, p-YAP, PKCι, p-PKCι, PKCζ and p-PKCζ was detected in tumor cells but not in stromal cells (Fig. 1). YAP, p-YAP, and p-PKCι staining exhibited both nuclear and cytoplasmic expression patterns (Fig. 1a, b, and d). PKCι, PKCζ and p-PKCζ staining exhibited only cytoplasmic expression without a membranous or nuclear staining pattern (Fig. 1c, e, and f). Immunohistochemical expression (nuclear or cytoplasmic) of YAP, p-YAP, PKCι, p-PKCι, PKCζ and p-PKCζ proteins were all counted and evaluated with clinicopathologic parameters. Nuclear YAP, cytoplasmic p-YAP, cytoplasmic PKCι, cytoplasmic p-PKCι, cytoplasmic PKCζ, and cytoplasmic p-PKCζ were selectively assessed that those expressions showed a significant relation with clinicopathologic parameters.

Of 200 LAC tissue samples examined protein expression by immunohistochemistry and expression statuses were summarized in Additional file 1: Tables S1, S2, and S3. The YAP (+) and p-YAP (+) was positive in 27% (54/200), YAP (+) and p-YAP (−) was positive in 21% (41/200), YAP (−) and p-YAP (+) was positive in 22% (43/200), YAP (−) and p-YAP (−) was positive in 31% (62/200). YAP was positive in 48% (95/200) and p-YAP was positive in 49% (97/200). The PKCι (+) and p-PKCι (+) was positive in 13% (25/200), PKCι (+) and p-PKCι (−) was positive in 9% (17/200), PKCι (−) and p-PKCι (+) was positive in 25% (49/200), and PKCι (−) and p-PKCι (−) was positive in 55% (109/200). PKCι was positive in 21% (42/200) and p-PKCι was positive in 37% (74/200). The PKCζ (+) and p-PKCζ (+) was positive in 35% (69/200), PKCζ (+) and p-PKCζ (−) was positive in 25% (50/200), PKCζ (−) and p-PKCζ (+) was positive in 6% (12/200), and PKCζ (−) and p-PKCζ (−) was positive in 35% (69/200). PKCζ was positive in 60% (119/200), and p-PKCζ was positive in 41% (81/200).

When separately assessed expression pattern of each immunostaining, nuclear YAP protein expression was correlated with cytoplasmic p-YAP expression (*p* = 0.000) (Table 1). Cytoplasmic p-YAP was positively correlated with cytoplasmic PKCι, cytoplasmic PKCζ, cytoplasmic p-PKCι, and cytoplasmic p-PKCζ immunohistochemical protein expression (*p* < 0.05, all). The immunohistochemical protein expression levels of cytoplasmic PKCι and PKCζ were positively correlated with those of p-PKCι and p-PKCζ (p < 0.05, all). Dual staining of PKCι and PKCζ revealed co-expressed cytoplasmic pattern of both antibodies (Additional file 2: Figure S1).

**Table 1:** Correlation between YAP, p-YAP, PKCι, p-PKCι, PKCζ, and p-PKCζ immunohistochemical expression in LAC

Spearman’s Rho		p-YAP	PKCι	p-PKCι	PKCζ	p-PKCζ
YAP	Correlation coefficient	0.258**	0.044	−0.002	0.117	0.024
	Sig. (2-tailed)	0.000	0.537	0.972	0.099	0.737
p-YAP	Correlation coefficient		0.537*	0.431**	0.297**	0.443**
	Sig. (2-tailed)		0.015	0.000	0.000	0.000
PKCι	Correlation coefficient			0.291**	0.243**	0.364**
	Sig. (2-tailed)			0.000	0.001	0.000
p-PKCι	Correlation coefficient				0.343**	0.600**
	Sig. (2-tailed)				0.000	0.000
PKCζ	Correlation coefficient					0.499**
	Sig. (2-tailed)					0.000

** *p* < 0.001

### Associations between YAP, p-YAP, PKCι, p-PKCι, PKCζ and p-PKCζ immunohistochemical expression levels and clinicopathological variables, including patient survival

A total of 200 LAC patient tissue samples were investigated for clinicopathological variables. The patients’ ages ranged from 36 to 91 years, with a mean age of 65.5 years. Male patients were slightly predominant to women (male:female = 1.1:1). Seventy-six patients had a smoking history (mean pack-years of smoking = 11 years). EGFR mutations with TKI sensitivity were detected in 80 patients. EGFR-TKI-resistant mutations were not identified.

The YAP, p-YAP, PKCι, p-PKCι, PKCζ and p-PKCζ protein expression levels were analysed in relation to clinicopathological features. Among these proteins, P-PKC*ι* immunohistochemical expression was significantly correlated with a higher pathological stage (I-II vs. III-IV), an acinar pattern (lepidic vs. acinar), and distant metastasis (*p* = 0.039, *p* = 0.005, and *p* = 0.028, respectively) (Table 2). P-PKCζ immunohistochemical expression was positively correlated with an acinar pattern (acinar vs. lepidic) (*p* = 0.000). PKCι, PKCζ, YAP and p-YAP immunohistochemical expression levels were not correlated with clinicopathological features.

**Table 2:** Correlation between p-PKCι and p-PKCζ immunohistochemical expression and the clinicopathological features of LAC patients

Characteristics	Patients		p-PKCι		Patients		p-PKCζ	
	No. (%)	Low	High	P	No. (%)	Low	High	P
Sex				0.736				0.880
Male	105 (53)	65 (52)	40 (54)		105 (53)	63 (53)	42 (52)	
Female	95 (48)	61 (48)	34 (46)		95 (48)	56 (47)	39 (48)	
Age				0.061				0.777
< 60	59 (30)	43 (34)	16 (22)		59 (26)	36 (30)	23 (28)	
≥ 60	141 (71)	82 (66)	58 (78)		141 (71)	83 (70)	58 (72)	
Pathological stage				0.039				0.423
I-II	168 (84)	111 (88)	57 (77)		168 (84)	102 (86)	66 (82)	
III-IV	32 (16)	15 (12)	17 (23)		32 (16)	17 (14)	15 (19)	
Histological subtype				0.005				0.000
Acinar	157 (79)	91 (72)	66 (89)		157 (79)	83 (70)	74 (91)	
Lepidic	43 (22)	35 (28)	8 (11)		43 (22)	36 (30)	7 (8)	
Distant metastasis				0.028				0.057
Absent	188 (94)	122 (97)	66 (89)		188 (94)	115 (97)	73 (90)	
Present	12 (6)	4 (3)	8 (11)		12 (6)	4 (3)	8 (10)	
Chemotherapy				0.061				0.618
Not done	130 (65)	88 (70)	42 (57)		130 (65)	79 (66)	51 (63)	
Done	70 (35)	38 (30)	32 (43)		70 (35)	40 (34)	30 (37)	
EGFR				0.439				0.637
Wild	106 (57)	63 (55)	43 (61)		106 (57)	59	47	
Mutant	80 (43)	52 (45)	28 (39)		80 (43)	47	33	

EGFR, Epidermal growth factor receptor; Chemotherapy, Post-surgical chemotherapy

**Fig. 2:**
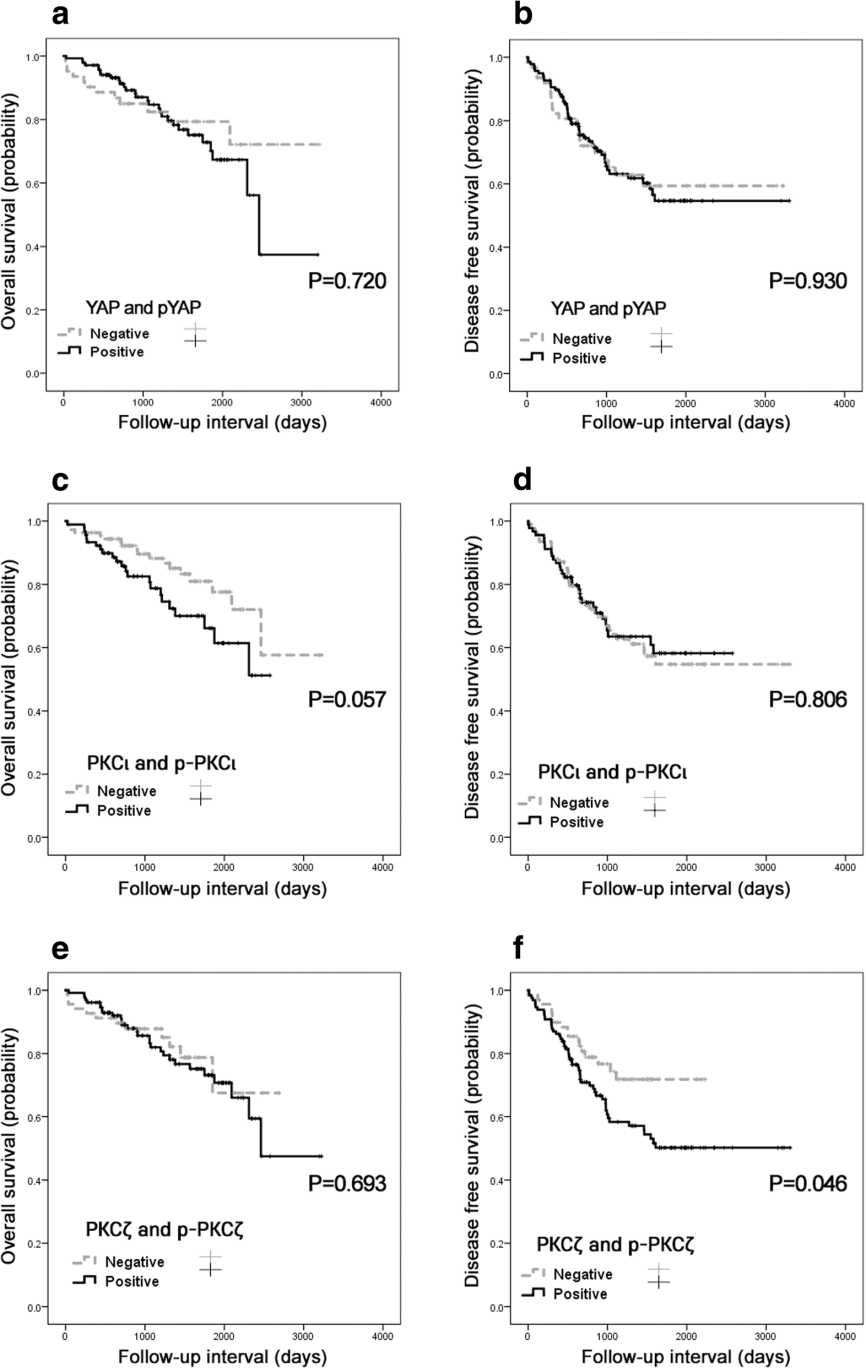
Kaplan-Meier curves according to immunohistochemical statu of (**a**) YAP/p-YAP, (**c**) PKCι/p-PKCι, and (**e**) PKCζ/p-PKCζ: overall survival and (**b**) YAP/p-YAP, (**d**) PKCι/p-PKCι, and (**f**) PKCζ/p-PKCζ: disease-free survival

Overall and disease-free survival analyses were performed with 200 LAC patients. The Kaplan-Meier survival curves and log-rank tests were evaluated with expression status (Fig. 2). YAP (aPKC) or pYAP (p-aPKC) positive considered “positive” and YAP and p-YAP (aPKC and p-aPKC) negative was considered “negative” (Fig. 2a and b). The Kaplan-Meier survival curves showed a tendency of positive relation with PKC*ι* immunohistochemical expression and shortened overall survival (Fig. 2c; *p* = 0.057) and a significant association between positive PKCζ immunohistochemical expression and shortened disease free survival (Fig. 2f; *p* = 0.046).

**Fig. 3:**
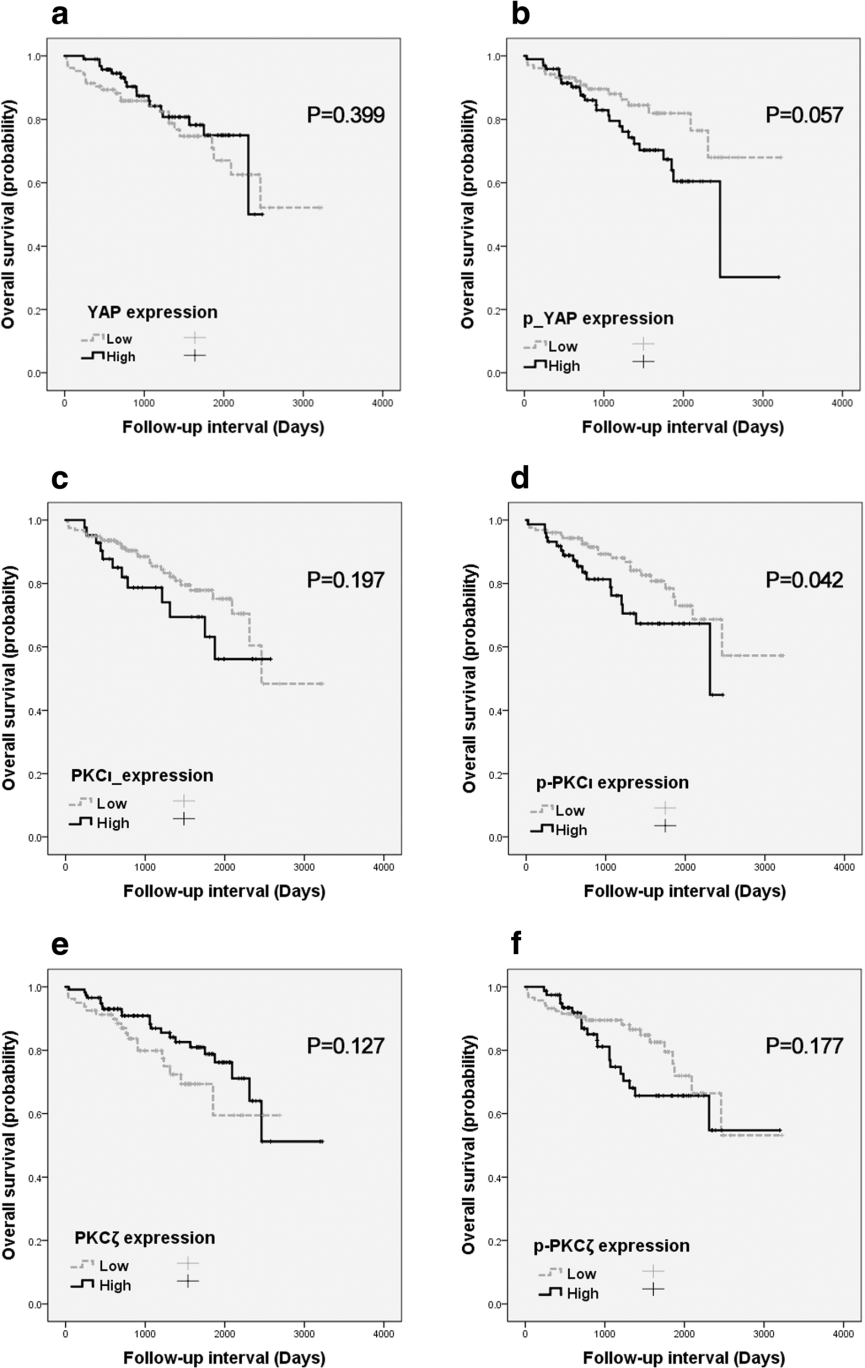
Kaplan-Meier curves according to (**a**) YAP, (**b**) p-YAP, (**c**) PKCι, (**d**) p-PKCι, (**e**) PKCζ, or (**f**) p-PKCζ immunohistochemical expression in LAC (*n* = 200): overall survival

YAP, p-YAP, PKCι, p-PKCι, PKCζ and p-PKCζ protein expression was separately evaluated with patient survival (Fig. 3). The Kaplan-Meier survival curves showed a significant association between high p-PKC*ι* immunohistochemical expression and shortened overall survival (Fig. 3d; *p* = 0.042). YAP, p-YAP, PKCι, PKCζ, and p-PKCζ did not have prognostic implications for patient overall survival (*p* = 0.399, p = 0.057, *p* = 0.197, *p* = 0.127, and *p* = 0.177, respectively). The multivariate analysis using the Cox proportional hazard model was performed with age, T-stage (1&2 vs 3&4), distant metastasis, recurrence, and p-PKCι expression (Additional file 1: Table S4). p-PKCι immunohistochemical expression did not reach statistical significance for overall survival in the multivariate analysis (*p* = 0.134).

### YAP, PRKCI, and PRKCZ mRNA levels between LAC and non-neoplastic lung tissue samples

**Fig. 4:**
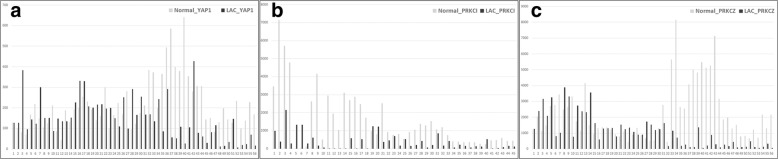
YAP, PRKCI, and PRKCZ mRNA levels in LAC with paired non-neoplastic lung tissues; (**a**) YAP1, (**b**) PRKCI, and (**c**) PRKCZ (*n* = 56, *n* = 45, *n* = 56, respectively)

YAP, PRKCI, and PRKCZ mRNA expression was evaluated in pairs of LAC and matched non-neoplastic lung tissues (Fig. 4). The YAP, PRKCI and PRKCZ mRNA levels were examined by qRT-PCR, and the relative quantitation level was determined. The YAP mRNA expression levels in the LAC tissues were higher in 24 (43%) out of 56 cases than in the non-neoplastic tissues. The PRKCI mRNA expression levels in the LAC tissues were higher in 6 (13%) out of 45 cases than in the non-neoplastic tissues. The PRKCZ mRNA expression levels in the LAC tissues were higher in 20 (36%) out of 56 cases than in the non-neoplastic tissues. The YAP and PRKCZ mRNA levels in the LAC tissues were positively correlated with each other (*p* = 0.000) (Table 3). The YAP and PRKCI mRNA levels and the PRKCI and PRKCZ mRNA levels were not correlated with each other (*p* = 0.120 and *p* = 0.562).

**Table 3:** Correlation between YAP, PRCKI, and PRKCZ mRNA levels in LAC

Spearman’s Rho		PRKCI	PRKCZ
YAP	Correlation coefficient	0.228	0.484**
	Sig. (2-tailed)	0.120	0.000
PRKCI	Correlation coefficient		−0.089
	Sig. (2-tailed)		0.562

** *p* < 0.001

**Fig. 5:**
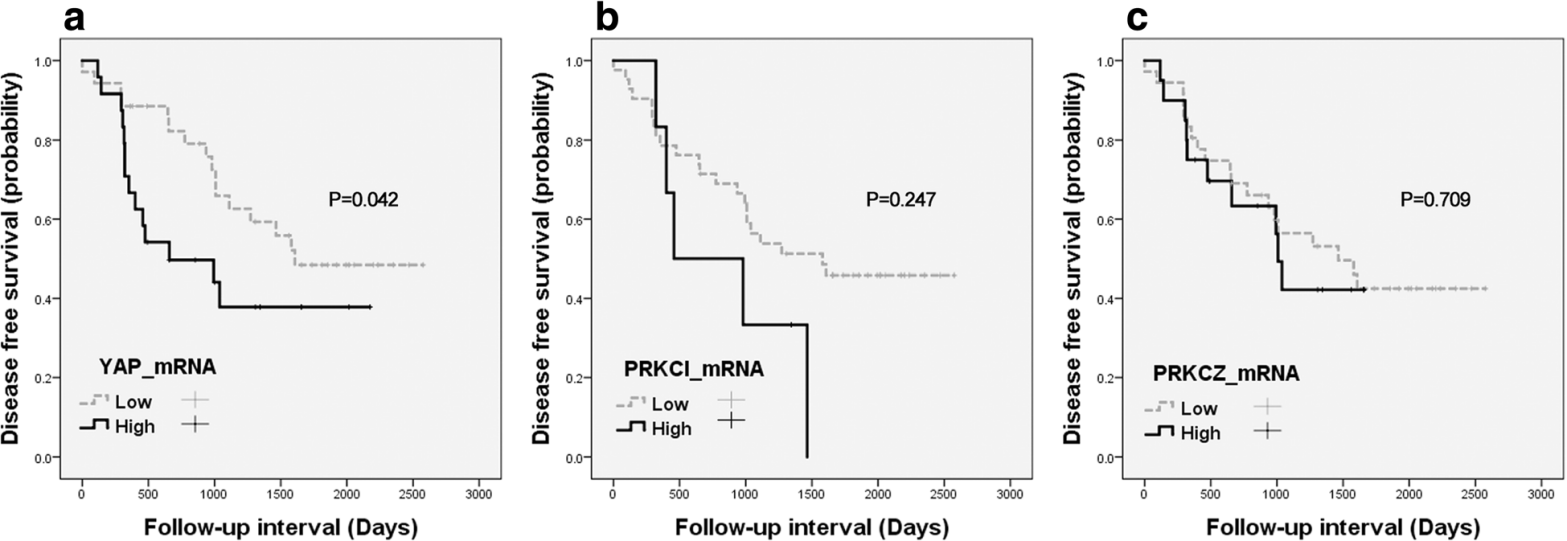
Kaplan-Meier curves according to the (**a**) YAP, (**b**) PRKCI or (**c**) PRKCZ mRNA level in LAC (*n* = 60): disease-free survival

The associations between the YAP, PRKCI, and PRKCZ mRNA levels and the clinicopathological characteristics, including disease-free survival, were evaluated. High YAP mRNA levels were correlated with a higher pathological stage (*p* = 0.010) (Table 4). High PRKCZ was associated with a higher pathological stage (*p* = 0.035). High PRKCI was related to wild-type EGFR (*p* = 0.016). The Kaplan-Meier survival curves and log-rank tests showed a significant association between high YAP mRNA levels and shortened disease-free survival (*p* = 0.042) (Fig. 5).

**Table 4:** Correlation between YAP, PRKCI, and PRKCZ mRNA levels and the clinicopathological features of LAC patients

Characteristics	Patients		YAP		Patients		PRKCI				PRKCZ	
	No. (%)	Low	High	P	No. (%)	Low	High	P	No. (%)	Low	High	P
Sex				0.026				0.445				0.140
Male	30 (51)	22 (63)	8 (33)		25 (52)	21 (50)	4 (67)		29 (52)	16 (44)	13 (65)	
Female	29 (49)	13 (37)	16 (67)		23 (48)	21 (50)	2 (33)		27 (48)	20 (56)	7 (35)	
Age				0.143				0.788				0.081
< 60	13 (22)	10 (29)	3 (13)		10 (21)	9 (21)	1 (17)		13 (23)	11 (31)	2 (10)	
≥ 60	46 (78)	25 (71)	21 (88)		38 (79)	33 (79)	5 (83)		43 (77)	25 (69)	18 (90)	
Pathological stage				0.010				0.592				0.035
I-II	52 (88.1)	34 (97)	18 (75)		43 (90)	38 (91)	5 (83)		49 (88)	34 (94)	15 (75)	
III-IV	7 (12)	1 (3)	6 (25)		5 (10)	4 (10)	1 (17)		7 (13)	2 (6)	5 (25)	
Histological subtype				0.803				0.877				0.088
Acinar	50 (85)	30 (86)	20 (83)		41 (85)	36 (56)	5 (83)		48 (86)	33 (92)	15 (75)	
Lepidic	9 (15)	5 (14)	4 (17)		7 (15)	6 (14)	1 (17)		8 (14)	3 (8)	5 (25)	
Distant metastasis				0.223				NA				0.176
Absent	58 (98)	35 (100)	23 (96)		42 (100)	42 (100)	6 (100)		55 (98)	36 (100)	19 (95)	
Present	1 (2)	0 (0)	1 (4)		0	0	0		1 (2)	0 (0)	1 (5)	
Chemotherapy				0.342				0.445				0.625
Not done	35 (59)	19 (54)	16 (67)		25 (52)	21 (50)	4 (67)		34 (61)	21 (58)	13 (65)	
Done	24 (41)	16 (46)	8 (33)		23 (48)	21 (50)	2 (33)		22 (39)	15 (42)	7 (35)	
EGFR				0.853				0.016				0.836
Wild	33 (57)	19 (56)	14 (58)		26 (54)	20 (48)	6 (100)		32 (58)	20 (57)	12 (60)	
Mutant	25 (43)	15 (44)	10 (42)		22 (46)	22 (52)	0 (0)		23 (42)	15 (43)	8 (40)	

EGFR, Epidermal growth factor receptor; Chemotherapy, Post-surgical chemotherapy; NA, not applicable

The correlation between mRNA level and immunohistochemical protein expression was evaluated. YAP and p-YAP protein expression paralleled YAP1 mRNA expression (*p* = 0.005 and *p* = 0.001). PKCι and p-PKCι protein expression did not parallel PPRKCI mRNA expression (*p* = 0.420 and *p* = 0.301). PKCζ and p-PKCζ protein expression did not parallel PRKCZ mRNA expression (*p* = 0.052 and *p* = 0.385).

### YAP modulates PKCι, p-PKCι, PKCζ and p-PKCζ expression in LAC cell lines

**Fig. 6:**
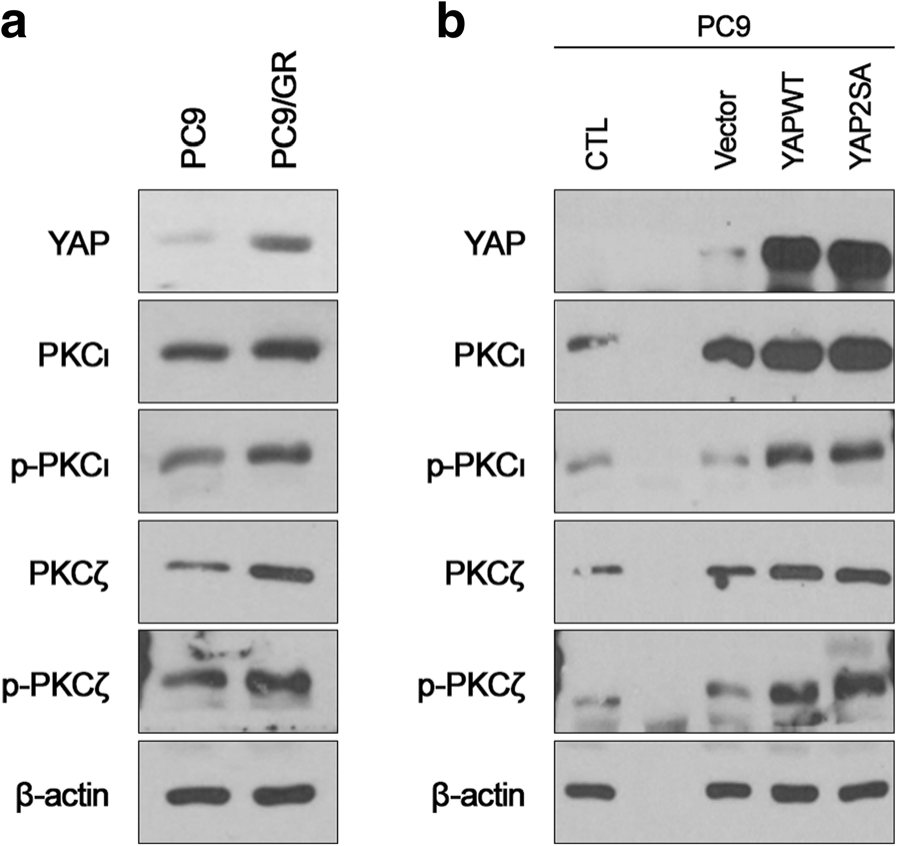
YAP, PKCι, p-PKCι, PKCζ, and p-PKCζ protein expression in LAC cell lines (**a**) Western blot analysis of PC9 and PC9/GR (**b**) Increased PKCι, p-PKCι, PKCζ, and p-PKCζ expression following YAP overexpression in the PC9 cell line

The PKCι, p-PKCι, PKCζ, and p-PKCζ expression levels were compared between the PC9 and PC9/GR LAC cell lines. YAP expression was higher in PC9/GR cells than in PC9 cells. PC9/GR cells also showed higher PKCι, p-PKCι, PKCζ, and p-PKCζ expression than PC9 cells (Fig. 6a). To validate a role for YAP in the regulation of PKCι, p-PKCι, PKCζ and p-PKCζ protein expression, YAP levels were modulated. YAP induced PKCι and PKCζ and contributed significant phosphorylation of PKCι and PKCζ proteins. Higher induction of p-PKCι and p-PKCζ were also identified in the aggressiveness of LAC (Fig. 6b).

## Discussion

Since the identification of PKC as a major cellular receptor for tumor-promoting phorbol esters 35 years ago, the PKC enzymes have been implicated in tumorigenesis and cancer progression. Both PKCι and PKCζ consist of aPKC isozymes and are known to have 70% homology in functional domains. However, PKCι and PKCζ are believed to play different roles in tumorigenesis. Some studies have suggested that PKCι behaves as an oncogenic factor and that PKCζ serves a tumor suppressive role [16, 17]. Elevated or amplified PKCι expression has been observed in cancers and is related to poor prognostic factors [6]. PKCζ has been shown to be up- or downregulated in various cancers, with conflicting clinical significance [18–21].

In this study, positive PKCι protein levels in LAC patients showed a tendency of relation to poor overall survival. When phosphorylation status of aPKC proteins were separately evaluated that upregulated p-PKCι protein levels in LAC patients were significantly correlated with higher pathological stage and shortened overall survival. Positive PKCζ protein levels in LAC patients was significantly related to shortened disease free survival. Upregulated p-PKCζ protein levels of LAC patients did not show a clinical impact. P-PKCι protein expression was shown to be a better poor prognostic marker than that of p-PKCζ in LAC. Phosphorylation of aPKC is considered to be involved in distinct biological activities. An increase in phosphorylated-aPKC protein expression was observed in tumorigenesis and showed a relationship with prognostic significance [22–24].

PKCι and PKCζ and their relationship with YAP, a downstream effector of Hippo, were evaluated in LAC cell lines. YAP Aggressive LAC (PC9/GR) cell lines showed elevated PKCι, p-PKCι, PKCζ and p-PKCζ protein levels with concomitantly elevated YAP levels compared to PC9 cell lines [25]. In previous studies, knockdown of PKCι and PKCζ led to a decrease in nuclear YAP expression [26, 27]. The oncogenic role of YAP has been revealed in various cancers [28, 29], whereby upregulated YAP mRNA levels are related to higher pathological stages and shortened disease-free survival in LAC. YAP induced aPKC upstream receptor proteins. aPKC protein expression was positively correlated with YAP overexpression in the LAC cell line study.

In LAC tissue samples, YAP mRNA and PRKCZ mRNA levels were positively correlated, but YAP mRNA and PRKCI mRNA levels were not positively correlated. aPKC expression at the transcriptional level did not parallel that at the protein level, and this discrepancy in relation to the clinical significance of the mRNA and protein levels was evident in this study. In addition, high mRNA levels of PKCι were observed more often in normal tissues than in LAC tissues. Not depending on production of activity of mRNA level, but localization of the aPKC protein in tumorigenesis or under external stimulation must be considered to evaluate the role of aPKC in cancers [30, 31]. Especially overexpression of YAP contributed upregulation of phosphorylation of PKCι, and PKCζ in the PC9 cell line. Archibald et al. proposed that membranous PKCζ inactivates Hippo signalling. PKCζ and the related protein complex phosphorylates Hippo/YAP and leads to degradation [32]. Translocation of or a protein-protein interaction with PKCζ is suggested to release YAP for nuclear accumulation and increase cellular proliferation [13]. Functional modulation of aPKCs in LAC is not simply regulated at the transcriptional level. In addition, Rac1/Mek/Erk-, Smoothened/GLI-, and NF-κB-dependent pathways have been suggested to function as effectors of the aPKC activation pathway, and different downstream effectors must also be considered to be related to the role of aPKC in cancers [6, 33–35].

## Conclusions

**Fig. 7:**
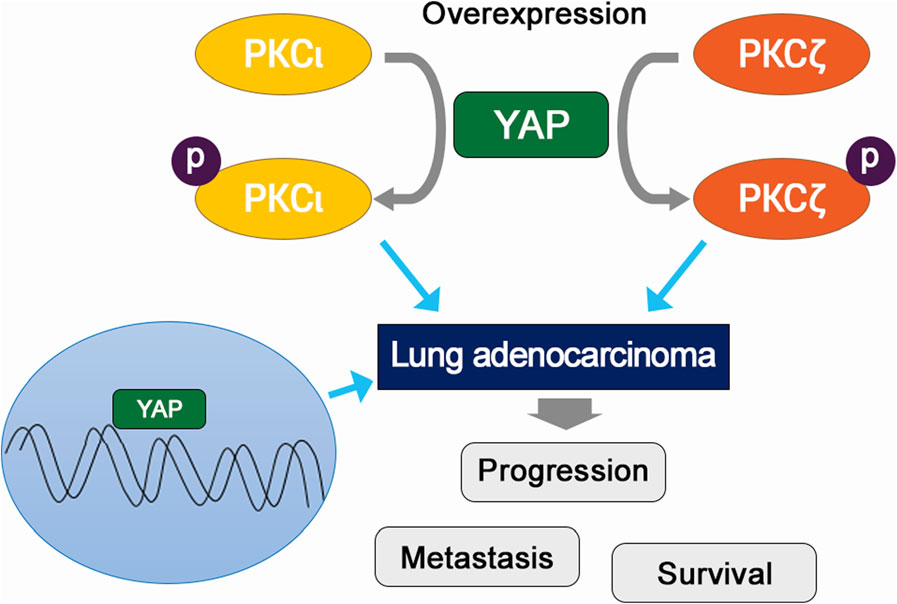
An illustration of the relationships of YAP, PKCι, and p-PKCι. YAP contributes generation of p-PKCι and p-PKCζ. Nuclear YAP, cytoplasmic of p-PKCι and p-PKCζ related with lung adenocaricnoma progression, metastasis, and poor patient survival

Herein, the expression pattern and clinical relevance of PKCι and PKCζ is evaluated in relation to YAP, a downstream effector of Hippo in LAC. Phosphorylation of PKCι and PKCζ are suggested to be related to YAP overexpression in LAC (Fig. 7). Both PKCι and PKCζ co-express in LAC and play distinct roles in LAC that p-PKCι overexpression is suggested to be associated with poor prognostic factors. Understanding the activation and functional differences between aPKC members helps to develop novel targets for LAC. Further investigation of the underlying mechanisms of aPKCs, especially p-PKCι, and related signalling pathways in LAC is required.

## Additional files

Additional file 1: **Table S1.** Comparison the number of YAP and pYAP immunohistochemical expression. **Table S2.** Comparison the number of PKCι and p-PKCι, immunohistochemical expression. **Table S3.** Comparison the number of PKCζ and p-PKCζ immunohistochemical expression. **Table S4.** Multivariate analysis of p-PKCι with overall survival. (DOCX 22 kb)

Additional file 2: **Figure S1.** Dual immunohistochemical expression of PKCι and PKCζ. Both negative (A,B) and co-positive expression of PKCι and PKCζ protein in LAC (C,D). (TIF 4637 kb)

## Data Availability

All data generated or analysed during this study are included in this article.

## Abbreviations

EGFR: Epidermal growth factor receptor
LAC: Lung adenocarcinoma
PKC: Protein kinase C
PKCζ: Atypical protein kinase zeta
PKCι: Atypical protein kinase iota
TKI: Tyrosine kinase inhibitor
