# Supplementary material for: Clinical significance of atypical protein kinase C (PKCι and PKCζ) and its relationship with yes-associated protein in lung adenocarcinoma
Source: BMC Cancer. 2019 Aug 14;19:804. doi: 10.1186/s12885-019-5992-7 (PMC6693135; doi:10.1186/s12885-019-5992-7)
Supplement: Supplementary file 1 — Table S1. Comparison the number of YAP and pYAP immunohistochemical expression. Table S2. Comparison the number of PKCι and p-PKCι, immunohistochemical expression. Table S3. Comparison the number of PKCζ and p-PKCζ immunohistochemical expression. Table S4. Multivariate analysis of p-PKCι with overall survival. (DOCX 22 kb) [file 12885_2019_5992_MOESM1_ESM.docx]

Table S1. The number of YAP and p-YAP immunohistochemical expression statuses in the lung adenocarcinoma

|  | YAP (-) | YAP (+) |
| --- | --- | --- |
| p-YAP (-) | 62 (31%) | 41 (21%) |
| p-YAP (+) | 43 (22%) | 54 (27%) |

Table S2. The number of PKCι and p-PKCι immunohistochemical expression statuses in the lung adenocarcinoma

|  | PKCι (-) | PKCι (+) |
| --- | --- | --- |
| p-PKCι (-) | 109 (55%) | 17 (9%) |
| p-PKCι (+) | 49 (25%) | 25 (13%) |

Table S3. The number of PKCζ and p-PKCζ immunohistochemical expression statuses in the lung adenocarcinoma

|  | PKCζ (-) | PKCζ (+) |
| --- | --- | --- |
| p- PKCζ (-) | 69 (35%) | 50 (25%) |
| p- PKCζ (+) | 12 (6%) | 69 (35%) |

Table S4. Multivariate analysis results for overall survival in lung adenocarcinoma patients (n = 200)

|  |  | Overall survival | | |
| --- | --- | --- | --- | --- |
|  |  | P | HR | 95% CI |
| p-PKCι (low vs. high) |  | 0.134 | 1.652 | (0.856-3.186) |
| Age (under 60 vs. over 60) |  | 0.054 | 2.414 | (0.985-5.914) |
| Stage (I+II vs. III+IV) |  | 0.331 | 1.387 | (0.717-2.683) |
| Distant metastasis |  | 0.006 | 3.812 | (1.455-9.987) |
| Recurrence |  | 0.010 | 2.402 | (1.236-4.668) |

HR, hazard ratio; CI, confidence index; Age unit (years)
