# Supplementary figures and images for: Clinical significance of atypical protein kinase C (PKCι and PKCζ) and its relationship with yes-associated protein in lung adenocarcinoma
Source: BMC Cancer. 2019 Aug 14;19:804. doi: 10.1186/s12885-019-5992-7 (PMC6693135; doi:10.1186/s12885-019-5992-7)

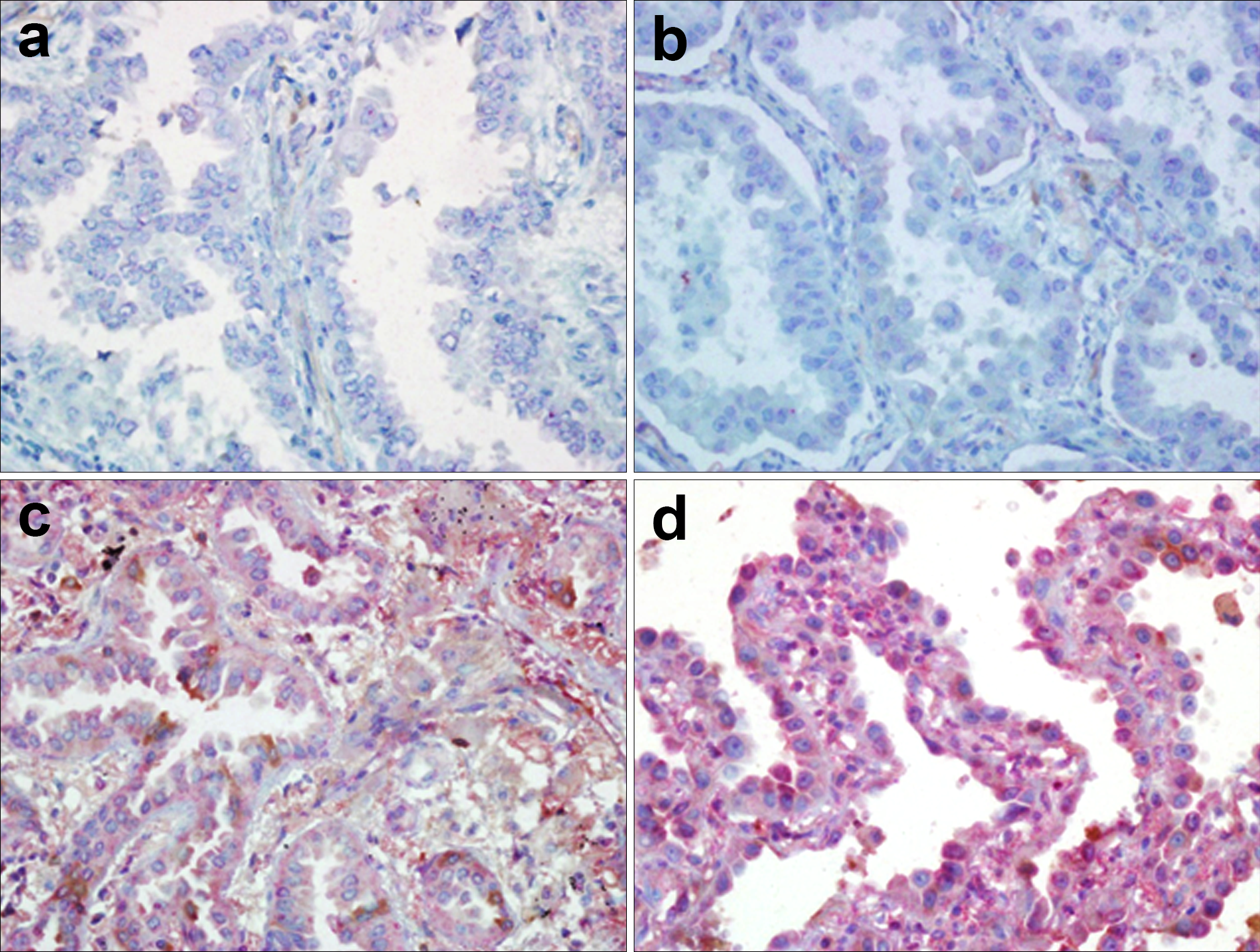

Supplement: Supplementary file 2 — Figure S1. Dual immunohistochemical expression of PKCι and PKCζ. Both negative (A,B) and co-positive expression of PKCι and PKCζ protein in LAC (C,D). (TIF 4637 kb) [file 12885_2019_5992_MOESM2_ESM.tif]
